# Identification and characterisation of thiamine pyrophosphate (TPP) riboswitch in *Elaeis guineensis*

**DOI:** 10.1371/journal.pone.0235431

**Published:** 2020-07-29

**Authors:** Atiqah Subki, Chai Ling Ho, Nur Farhah Nabihan Ismail, Aisamuddin Ardi Zainal Abidin, Zetty Norhana Balia Yusof

**Affiliations:** 1 Department of Biochemistry, Faculty of Biotechnology and Biomolecular Sciences, Universiti Putra Malaysia, Selangor, Malaysia; 2 Department of Cell and Molecular Biology, Faculty of Biotechnology and Biomolecular Sciences, Universiti Putra Malaysia, Selangor, Malaysia; 3 Laboratory of Marine Biotechnology, Institute of Bioscience, Universiti Putra Malaysia, Selangor, Malaysia; 4 Bioprocessing and Biomanufacturing Research Center, Universiti Putra Malaysia, Selangor, Malaysia; Auburn University College of Sciences and Mathematics, UNITED STATES

## Abstract

The oil palm (*Elaeis guineensis*) is an important crop in Malaysia but its productivity is hampered by various biotic and abiotic stresses. Recent studies suggest the importance of signalling molecules in plants in coping against stresses, which includes thiamine (vitamin B_1_). Thiamine is an essential microelement that is synthesized *de novo* by plants and microorganisms. The active form of thiamine, thiamine pyrophosphate (TPP), plays a prominent role in metabolic activities particularly as an enzymatic cofactor. Recently, thiamine biosynthesis pathways in oil palm have been characterised but the search of novel regulatory element known as riboswitch is yet to be done. Previous studies showed that thiamine biosynthesis pathway is regulated by an RNA element known as riboswitch. Riboswitch binds a small molecule, resulting in a change in production of the proteins encoded by the mRNA. TPP binds specifically to TPP riboswitch to regulate thiamine biosynthesis through a variety of mechanisms found in archaea, bacteria and eukaryotes. This study was carried out to hunt for TPP riboswitch in oil palm thiamine biosynthesis gene. Riboswitch detection software like RiboSW, RibEx, Riboswitch Scanner and Denison Riboswitch Detector were utilised in order to locate putative TPP riboswitch in oil palm *ThiC* gene sequence that encodes for the first enzyme in the pyrimidine branch of the pathway. The analysis revealed a 192 bp putative TPP riboswitch located at the 3’ untranslated region (UTR) of the mRNA. Further comparative gene analysis showed that the 92-nucleotide aptamer region, where the metabolite binds was conserved inter-species. The secondary structure analysis was also carried out using Mfold Web server and it showed a stem-loop structure manifested with stems (P1-P5) with minimum free energy of -12.26 kcal/mol. Besides that, the interaction of riboswitch and its ligand was determined using isothermal titration calorimetry (ITC) and it yielded an exothermic reaction with 1:1 stoichiometry interaction with binding affinities of 0.178 nM, at 30°C. To further evaluate the ability of riboswitch to control the pathway, exogenous thiamine was applied to four months old of oil palm seedlings and sampling of spear leaves tissue was carried out at days 0, 1, 2 and 3 post-treatment for expression analysis of *ThiC* gene fragment via quantitative polymerase chain reaction (qPCR). Results showed an approximately 5-fold decrease in *ThiC* gene expression upon application of exogenous thiamine. Quantification of thiamine and its derivatives was carried out via HPLC and the results showed that it was correlated to the down regulation of *ThiC* gene expression. The application of exogenous thiamine to oil palm affected *ThiC* gene expression, which supported the prediction of the presence of TPP riboswitch in the gene. Overall, this study provides the first evidence on the presence, binding and the functionality of TPP riboswitch in oil palm. This study is hoped to pave a way for better understanding on the regulation of thiamine biosynthesis pathway in oil palm, which can later be exploited for various purposes especially in manipulation of thiamine biosynthesis pathways in combating stresses in oil palm.

## Introduction

Thiamine (Vitamin B_1_) is a necessary microelement merited by its prominent role as a cofactor in some central metabolic activities such as in glycolysis and pentose phosphate pathways [1]. In recent years, thiamine has been designated to be related to plant protection studies. The active form of thiamine known as thiamine pyrophosphate (TPP) can directly control the *de novo* biosynthesis of thiamine through feedback regulation mechanism [2]. Other than its role as a cofactor, scientists are interested to divulge on the newly found role of this metabolite in plants as a signalling molecule during unfavourable conditions [3].

In Malaysia, palm oil industry is one of the important key economic drivers that secures substantial income for the national economy [4]. Nevertheless, environmental stresses have influenced the growth and its production. This issue has to be taken seriously because it has a major impact on the productivity of plants especially in oil palm. To further understand the metabolism that occur during the event, a rigorous study on the stressor effect towards plant regulation and how plants cope with the stress at genetic level is increasing in number.

Some comprehensive studies on the effects of biotic and abiotic stresses on the regulation of thiamine in oil palm have previously been done. For example, boosting thiamine content could increase plants’ resistance towards stresses [1]Furthermore, application of endophythic fungus upregulated the gene expression of genes involved in thiamine biosynthesis pathway thus increasing the total thiamine content in oil palm [5–7]. On the other hand, systemic acquired resistance (SAR) in several plants were shown to be induced upon thiamine application, suggesting role of thiamine as a stress-responsive molecule [1].

The regulation of thiamine biosynthesis pathways is uncommon from other type of vitamins. Previous studies by Guan *et al*. (2014) [8] revealed that the energy cost of thiamine synthesis was higher as compared to other vitamin co-factor, suggesting the presence of novel regulatory element called riboswitch. Riboswitch is an RNA molecule that allows direct binding of specific ligand to it, resulting in a change in protein production. The most studied class of riboswitch is TPP riboswitch [9]. The regulation of thiamine biosynthesis via riboswitch has been widely identified in prokaryotes, plants, and certain fungi [10].

TPP riboswitch mechanism is found to be significant in the maintenance of adequate thiamine levels in plants [11]. In response to the environmental changes, this mechanism causes the cells to sense the intracellular concentration of TPP metabolites and cause the conformational changes to occur which leads to several mechanisms of regulation to take place [12]. Generally, riboswitch function in themodulation of gene expression by executing transcription and splicing activity and can be considered as viable candidates for a sophisticated mechanism of regulatory control in RNA-based life can [13].

Evidently, the elucidation of thiamine biosynthesis and identification of TPP riboswitch have been widely conducted in other organisms but not in oil palm. Recently, the biosynthesis of thiamine in oil palm has been characterised, but to further understand the mechanism that occurs during the event, a rigorous study on how thiamine biosynthesis in oil palm is regulated should be conducted since there are very limited amount of studies involving thiamine in oil palm available, let alone the studies on TPP riboswitch [14] Although the total genome of oil palm is currently revealed, the utilisation of this information on localisation of novel regulatory element like riboswitch is yet to be done.

The core objectives in hunting for riboswitch elements is to further understand if riboswitch actually exist as an alternative system in regulating gene expression. The search of new novel RNA regulatory element like riboswitch is crucial to fully understand how thiamine is made as it has been seen as a important signalling molecule in modulating stresses in oil palm.

## Materials and methods

### *In-Silico* identification of TPP riboswitch

#### Data mining

Genomic data was obtained from National Centre for Biotechnology Information (NCBI) GenBank (https://www.ncbi.nlm.nih.gov/genbank/). *ThiC* gene sequence which encodes for hydroxymethylpyrimidine pyrophosphate (HMP-P) from plants and fungus namely *Arabidopsis Thaliana* (Accession no.: NC_003071.7), *Phoenix Dactylifera* (Accession no.: NW_008246537.1), *Malus Domestica* (Accession no. NC_024249.1) and *Neurospora crassa* (Accession no.: AY007661.1) were collected for reference and comparison purposes [15].

#### Riboswitches identification through web tools search and primer design

All collected sequences were subjected to riboswitch analysis software including RiboSW (http://ribosw.mbc.nctu.edu.tw/), Riboswitch Explorer (http://132.248.32.45/cgi-bin/ribex.cgi), Riboswitch Scanner (http://service.iiserkol.ac.in/~riboscan/application.html) and Denison Riboswitch Detector, DRD (http://drd.denison.edu/) [16–19]. The exon and intron regions were analysed by using RNA analyser (http://rnaanalyzer.bioapps.biozentrum.uni-wuerzburg.de/) [20]. The gene sequence and position of primers were attached in S1 Appendix.

The *ThiC* gene of oil palm was also subjected to riboswitch analysis software to confirm the localisation of putative TPP riboswitch [21]. Sets of primers were manually designed for the amplification of the targeted region containing the putative TPP riboswitch fragment (ThiC TPP 1 (F) 5’-GGTGTGGTCTTGTGTCTT-3’; ThiC TPP 1 (R) 5’-CGGCTACAGCATGAACAT-3’). The primers’ details used for amplification of putative TPP riboswitch was mentioned in S1 Table.

### Analysis of ligand binding of TPP riboswitch in oil palm

#### Preparation of plant materials and isolation of DNA

Five months old oil palm seedlings from the variety of *Tenera* (*Dura* x *Pisifera*) was obtained from Felda Enstek, Negeri Sembilan, Malaysia. It was acclimatized for one week under normal nursery practices (watered with 125 mL tap water at 4.00 pm and maintained in a shaded area with natural sunlight exposure). Spear leaves samples were collected and immediately stored in -80°C until further use. The total DNA was extracted using commercial kit Qiagen DNeasy Plant Mini Kit (Germany).

#### Polymerase Chain Reaction (PCR) and in *vitro* transcription analyses

Polymerase chain reaction (PCR) was performed using MyTaq^TM^ Red Mix (Bioline, USA) according to the manufacturer’s protocol. The synthesis of RNA involves a set of experimental methods including; plasmid linearization, RNA *in vitro* transcription and purification of synthesised RNA. These procedures require a purified DNA template containing a promoter, ribonucleotide-triphosphates, buffer system and appropriate amount of RNA polymerase. The synthesis of RNA was conducted using HiScribe^TM^ T7 Quick High Yield RNA Synthesis Kit (New England Biolabs, USA).

#### Analysis of TPP riboswitch-ligand interaction by Isothermal Titration Calorimetry (ITC)

Molecular interaction between riboswitch and its ligand was studied using Nano ITC (TA Instrument 5303/LI0007, New Castle). RNA was diluted in Ultra-pure water into the concentrations of 30 mM and 10 mM. 150 μL of 30 mM. 10 mM of TPP was also prepared and both RNA sample and TPP ligand were degassed prior analysis. 200 μL RNA was loaded inside the cell and and 52 μL was added inside titration syringe. Reaction condition was set up as follows; 2.02 μL of injection volume, 300 sec of injection interval with 25 injections number at temperature of 30°C. The heat change was observed and recorded. The raw heat produced was analysed using Launch NanoAnalyze (New Castle, USA). The binding activity between ligand and its micromolecule can be calculated using Eq 1.

$$Dissociation\;constant\frac{1}{Association\;constant\;\left(Ka\right)}\;(Kd)=Where\;Ka\;was\;derived\;from\;the\;gradient\;of\;slope\;from\;thermogram.$$ Eq 1

### *In-vivo* analysis: Effect of *ThiC* gene expression and its metabolite production upon application of exogenous thiamine

#### Application of thiamine on the oil palm seedlings and collection of samples

Four month old oil palm seedlings were obtained from Malaysian Palm Oil Board (MPOB). All the seedlings were acclimatized for one week under standard nursery practice before being subjected for treatments. The treatment plots were arranged in a randomized complete block design (RCBD). About 2 L of 50 mM thiamine-hydrochloride (T-HCl) (Millipore, USA) was prepared prior to thiamine treatment [22]. Drenching technique was used by applying 125 mL of T-HCl to the seedlings. Non-treated seedlings served as control during the set up. Destructive sampling was conducted post-treatment with time point of 0, 24, 48 and 72 hours. The spear leaf tissues were cut using sterilised knife, lightly washed in 5% Clorox and rinsed by using sterile distilled water. All the samples were immediately stored in -80°C until further use.

*Expression analysis of ThiC gene fragment using quantitative Polymerase Chain Reaction (qPCR)*. Quantitative polymerase chain reaction (qPCR) was performed using SensiFAST^TM^ SYBR No-ROX Kit (Bioline, USA) according to manufacturer’s protocol. The expression of the gene of interest was normalised against actin and tubulin gene (references) based on Vandesompele *et al*., (2012) [23]. Upon normalisation, the expression of *actin* and *tubulin* was indicated as zero giving the expression unit of thiamine biosynthesis gene, *ThiC* in relative quantity. Calculation of normalisation of gene expression was achieved using 2^-ΔΔCT^ method as described by Livak and Schmittgen, 2001 [24]. List of primers used for the analysis of *ThiC* gene expression upon application of exogenous thiamine is as shown in S2 Table.

#### Quantification of thiamine and its metabolite using High Performance Liquid Chromatography (HPLC)

*Preparation of crude extract*. For the quantification of thiamine and its derivatives, extraction was carried out from 2.5 g spear leaves of oil palm sample in 10 mL of 0.1 N HCl. The mixture was completely mixed using vortex and incubated at 37°C for 16 hours. To obtain the supernatant, the mixture was centrifuged at 7000 rpm for 20 mins. The supernatant was then collected and stored in **-**80°C prior further use.

*Analysis of metabolite through HPLC*: *Gradient elution setting*. Two mobile phases (methanol and sodium phosphate) were prepared to set the HPLC machine (Agilent Technologies, 1200 series, USA) (as mentioned in S2 Text). The column used during the study was C18 (SGE, 250 x 4.6 mm, optimal flow rate of 1 mL per min) and the detector is diode array. The chromatography setting was derived from Kamarudin *et al*. (2017) [25]. The analyte was eluted at flow rate of 1 mL/min with an injection volume of 20 μL. The excitation was set at 375 nm and emission at 435 nm. General information of chromatography setting is mentioned in S3 Text. Calibration of standard was done and the retention times were at minutes 5.726 and 8.550 for TPP and thiamine respectively (as shown in S1 Fig).

#### Statistical analysis

Statistical analyses were performed using student t-test and non-linear regression analysis software. The significant level was set at *p* value <0.05 using Statistical Package for Social Science (SPSS).

## Results and discussion

### *In-silico* analysis: Bioinformatics approach in locating TPP riboswitch

#### Identification of putative TPP riboswitch in oil palm

**Fig 1:**
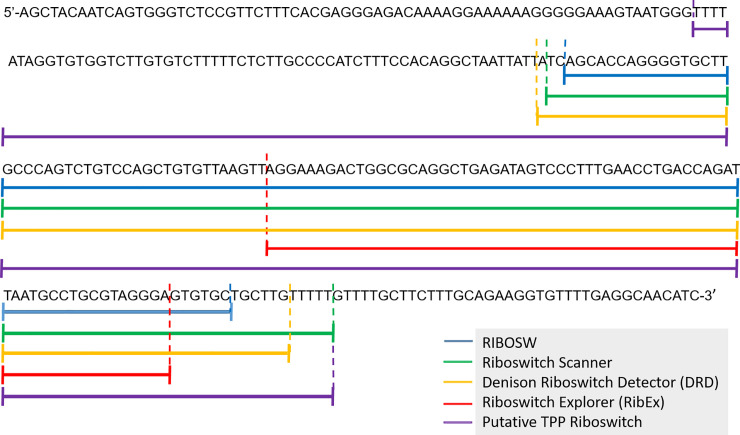
Comparison of riboswitch detection tools showed about 95% similarities of detection of putative TPP riboswitch sequences in *ThiC* gene of *Elaeis guineensis*. All tools tested detected the same region as a putative TPP riboswitch.

Identification of riboswitches was carried out through bioinformatics approaches. There were four riboswitch search engines used in this study namely Riboswitches Explorer (RibEx), RiboSW, Riboswitch Scanner and Denison Riboswitch Detector (DRD). The search analysis provided information in terms of its position, number of motif detected, structural prediction and the minimum free energy of structure. Different web based tools differ in terms of speed of performance (time of search computational analysis to complete), sensitivity and specificity (capture twice as much of information as the other) and the exclusive of the hits (percentage of coverage). A successful detection of putative TPP by all four tools used lies within the same position overlapping each other (Fig 1). Riboswitch Explorer could detect the shortest range of TPP motif while Riboswitch Scanner was able to detect the longest range of TPP motif in *ThiC* gene of *Elaeis guineensis*. A putative TPP riboswitch was detected in oil palm *ThiC* gene sequence when the sequences were subjected to the riboswitch detection software as mentioned earlier. The putative riboswitch is in the 3’ untranslated region (UTR) of the mRNA of oil palm with 192 nucleotides in length.

**Fig 2:**
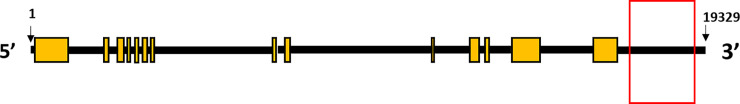
Illustration of DNA and mRNA of *Elaeis guineensis*’s whole *ThiC* gene depicting the exon (red) and intron (grey) regions. The putative TPP riboswitch was detected at the non-coding region of 15th intron nearly at the 3’ end of the mRNA sequence.

This result has been summarized and presented in Fig 2, which supports the previous finding on riboswitch as a short sequence ranging from 50 to 250 nucleotides in length [26]. TPP riboswitch has been previously found in *Arabidopsis thaliana* at the 3’-UTR of *ThiC* gene [27]. *Arabidopsis* sp. has been used as the central model organism in the riboswitch detection, since TPP riboswitch was extensively proven computationally and experimentally. A study by Mangel *et al*. (2017) [2] also revealed the presence of TPP riboswitches in 3’ UTR of *Cassava* sp. through sequence homology method.

Theoretically, riboswitch was expected to be present in the non-coding region of the mRNA [28]. For that reason, *ThiC* mRNA sequence of oil palm has been analysed by using RNA analyser to identify whether *ThiC* riboswitch falls within an intron region of the gene sequence. The putative TPP riboswitch in oil palm can be found in the intron region of the *ThiC* gene sequence. It is located nearly at the 3’ end of the gene sequence. This finding is in line with the study by Bocobza and Aharoni (2014) [3] where the TPP riboswitch was found to be located at the 3’ UTR of *ThiC* gene in 13 types of flowering plant. However, Li and Breaker (2013) [29] found the TPP riboswitch in *Neurospora crassa* to be located in the 5’ end of intron region, which is similar to the riboswitch character in bacteria.

This study focused on the search of TPP riboswitch in *ThiC* gene coding for the key enzymes in biosynthesis of thiamine. The primitive plant such as *Poa secunda* (bluegrass) from an ancient taxon contained riboswitches in both *ThiC* and *thi1* gene but through evolution only *ThiC* gene remained [30]. This strongly suggests that TPP riboswitch in *ThiC* gene plays a major role in regulating the synthesis of thiamine. The fact that it is located at the initial branch for the synthesis of the pyrimidine moiety also suggests how the thiamine biosynthesis pathway is tightly regulated in oil palm [31].

#### Comparative analysis of riboswitch web tools

The analysis of algorithm performed by each software was studied in terms of efficiency of searching homologous instances of known riboswitch, its sensitivity in detecting different kinds of riboswitches, output processing speed and size of the input that can be inserted at a time. The minimum free energy and formation of loop pattern for putative TPP riboswitch constructed were determined. There are three approaches utilised by softwares to detect riboswitches which are 1) identification of conserved stem-loop features which reside in the query sequence, 2) identification of aptamer region which is a highly conserved sequence in a particular class of riboswitch and 3) characterization of a riboswitch family using probabilistic model such as hidden markov model (HMM) [18,19]. Riboswitch scanner and RiboSw utilise the first and third approaches which the algorithm identify the query sequence riboswitches structural conformations and consensus sequence to produce HMM model [16,18]. However, Riboswitch scanner algorithm is much more updated as it utilises 5-fold cross-validated HMM models known as HMMER3. RibEx and DRD software utilised the second approach where RibEx utilises Cluster of Orthologous Groups (COG) database from the GenBank to identify riboswitch motif from the query and DRD operates by breaking the query sequence into overlapping smaller sequences which then align against consensus sequence of the riboswitch class using dynamic programming [17,19].

**Fig 3:**
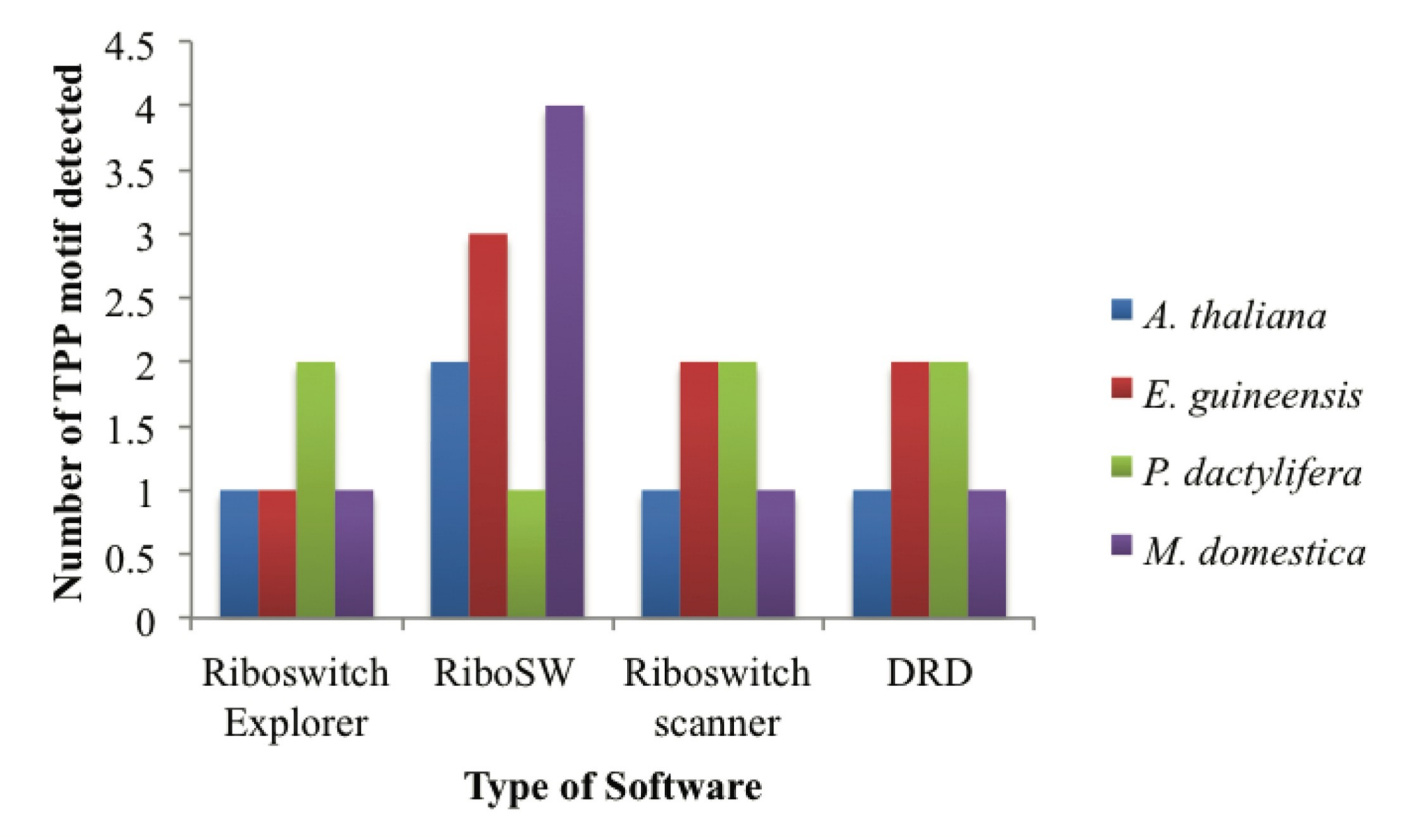
The number of TPP motif(s) detected using four different web tools tested on different plants that contained TPP riboswitch in their respective *ThiC* gene.

As summarised in Fig 3, Riboswitch Scanner and DRD were the two tools that provided highest hit similarity in TPP motif search with only one or two base pairs difference. Although RibEx, Riboswitch Scanner and DRD basically produced the same number of motifs detected, the region detected by RibEx showed a little variation in its position. Despite that, the detection was still at the same sequence region. RiboSW was the only tool that gave out the least similar number of TPP riboswitch detection. In terms of sensitivity, RiboSW was found to be the most sensitive since it can detect the most motif number. This data however does not support the relative sensitivity test of riboswitch tools conducted by Havill which claimed that DRD was the most current and sensitive tool among all with number of hits of 46 and relative sensitivity value of 0.90 [19]. In this study, RiboSW had 32 TPP riboswitch hits with TPR value of 0.64, which showed that the sensitivity value of this tool was lower than DRD tool. The TPR value for Riboswitch Scanner and RibEx is yet to be evaluated.

For region detection specificity, all tools tested managed to detect at least one motif that fell within the same range of region. In terms of speed of detection, all tools used provided an output display of <1 min. Although DRD algorithm was developed exclusively for bacterial genome scale, the principle was also applicable to eukaryotic data. When homologous domains *ThiC* and/or *ThiM* genes of eukaryotes were compared with *E*. *coli* (Gram negative) and *Clostridium acetobutylicum* (Gram positive), the consensus region of the sequence can still be displayed [29]. Different number of motifs detected by certain tool suggests that there is a wide spread distribution and diversity of their location which might be related to each other in performing the mechanism of regulation.

#### Analysis of secondary structure prediction of putative TPP riboswitch

Structural comparison was checked and analysed based on different software used. Putative TPP riboswitch constructed was checked for the minimum free energy and the ability to form hairpin loop structure. These are then extended by additional BLAST search. BLAST search on the initial riboswitch sequence produces known locations in various genomes that are familiar for the known aptamer at hand [8].

**Fig 4:**
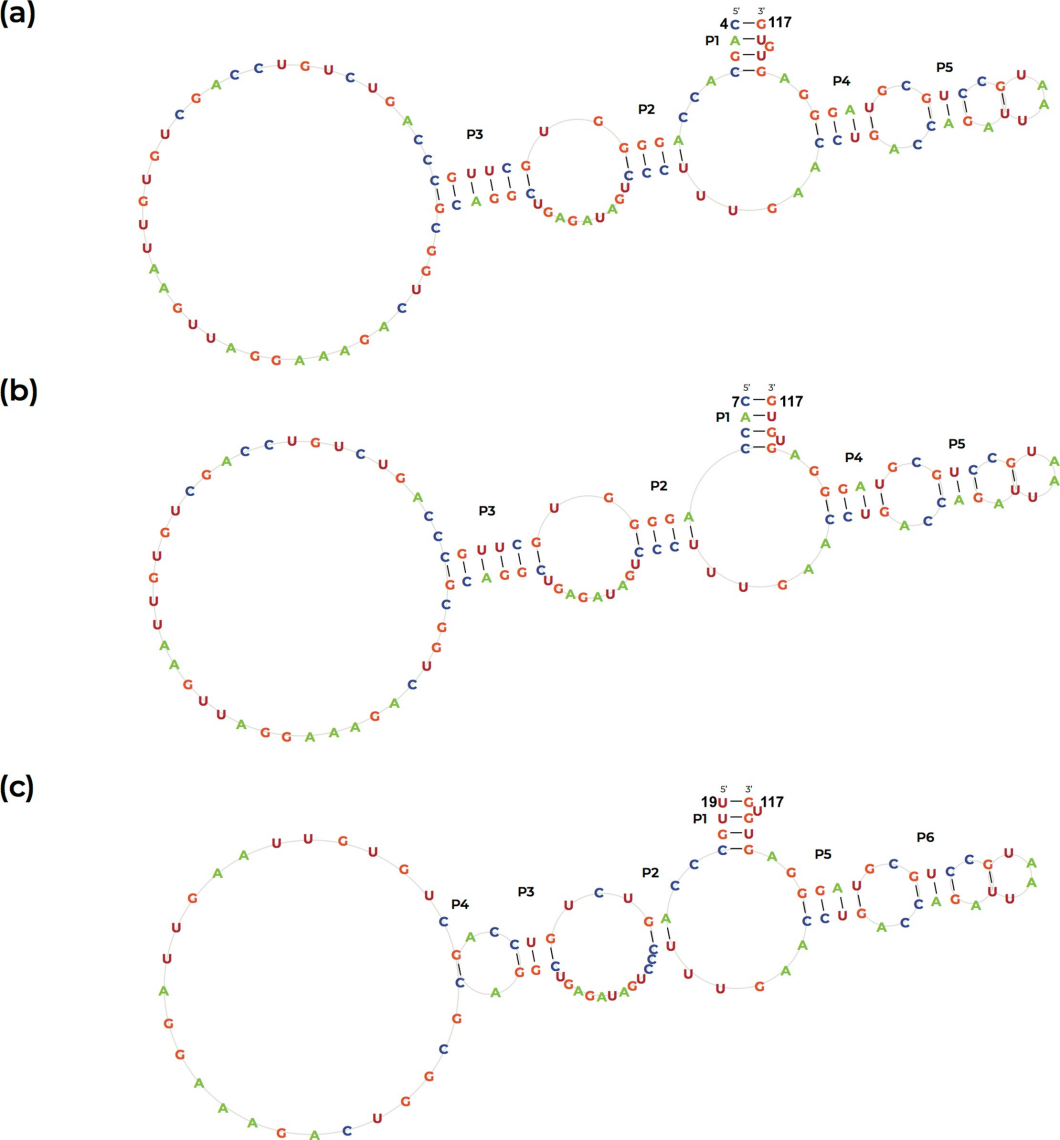
Proposed secondary structure of the putative TPP riboswitch in *Elaeis guineensis* with five stems, P1, P2, P3, P4 and P5 (in direction of 5’-3’) together with five loops predicted using RNA fold with minimum free energy of (a)-11.86 kcal/mol (b) -12.26 kcal/mol. and (c) 6.99 kcal/mol. There are three predicted structure produced by RiboSW software with four color codes representing the four bases.

The secondary-structure model was built for the promising riboswitch motifs. Although *ThiC* sequence of oil palm could be directly examined for TPP motif using riboswitch web interfaces, other selected plants that contained TPP riboswitch were aligned using pair wise sequences alignment for homology region detection particularly between *Elaeis guineensis* and selected plants. The consensus region that already contain TPP riboswitch motif was compared with the *ThiC* gene of *Elaeis guineensis* in order to further justify the result provided by web interface used. Some variation of predicted secondary structure of putative TPP riboswitches in *ThiC* gene of oil palm are as in (Fig 4A, 4B and 4C). Most secondary structure are similar but not the same because of sequence size variation from one to another.

Positional covariation had predicted the secondary structure, consisted of five stems which were P1 stem (CAGC), P2 stem (AGGGG), P3 stem (GCUUGC), P4 stem (CCUG), and P5 stem (CAG). G-U pairing, also known as wobble pairing could be seen in stem 1, 3 and 4. Wobble pairing is the base pairing of two nucleotides that does not follow the theoretical Watson-Crick base pairing rule. P2 forward stem is conserved when compared to TPP riboswitch of *Arabidopsis thaliana* while P3 is the most variable stem among all stem across various plant species [32]. The similar number of stems in the secondary structure of TPP riboswitches in plants and bacteria supports the fact that the sequence is highly conserved across species [32 and 33]. In spite of that fact, this kind of pairing is thermodynamically favourable than the Watson-Crick. Formation of five helixes occurred in conjunction of the presence of five stems. Helix happened from two sets of sequences that are not identical and is not a complementary base pair. This is in contrast with the stem formation which entails a complementary base pairing activity.

Although there were a numbers of secondary structures generated as an output during the analysis, the structure shown in Fig 4(B) was proposed to be structure for TPP riboswitch in *Elaeis guineensis* with minimum free energy of -12.26 kcal/mol. Minimum free energy is the total energy of all the loops, which depends on the degree and size of the loop [34]. The putative TPP riboswitch can fold itself into various structures but the most accurate structure would be the one that gives out the minimum free energy [32]. In fact, Free energy minimization not only the most stable, but also the most probable one in thermodynamic equilibrium [35]. Previous study mentioned the minimum free energy of secondary structure of putative riboswitches in many flowering plants and microorganisms ranged between -1.0 to -25.0 kcal/mol [32 and 35]. Based on Tinoco-Uhlenbeck postulate, although the energy calculation was made independent for each stem loop in the secondary structure folding, the endmost value indicates the sum of free energy for all base pairs. The determination of minimum free energy for each predicted structure is very important as for the stability of the complementary region. A variety of structures from various organisms have been uncovered and it is proposed that the regulation may be more extensive than the current finding [36]. Comparative gene analysis has been widely used in the study of gene function as it can determine in high percentage of correct structure.

**Fig 5:**
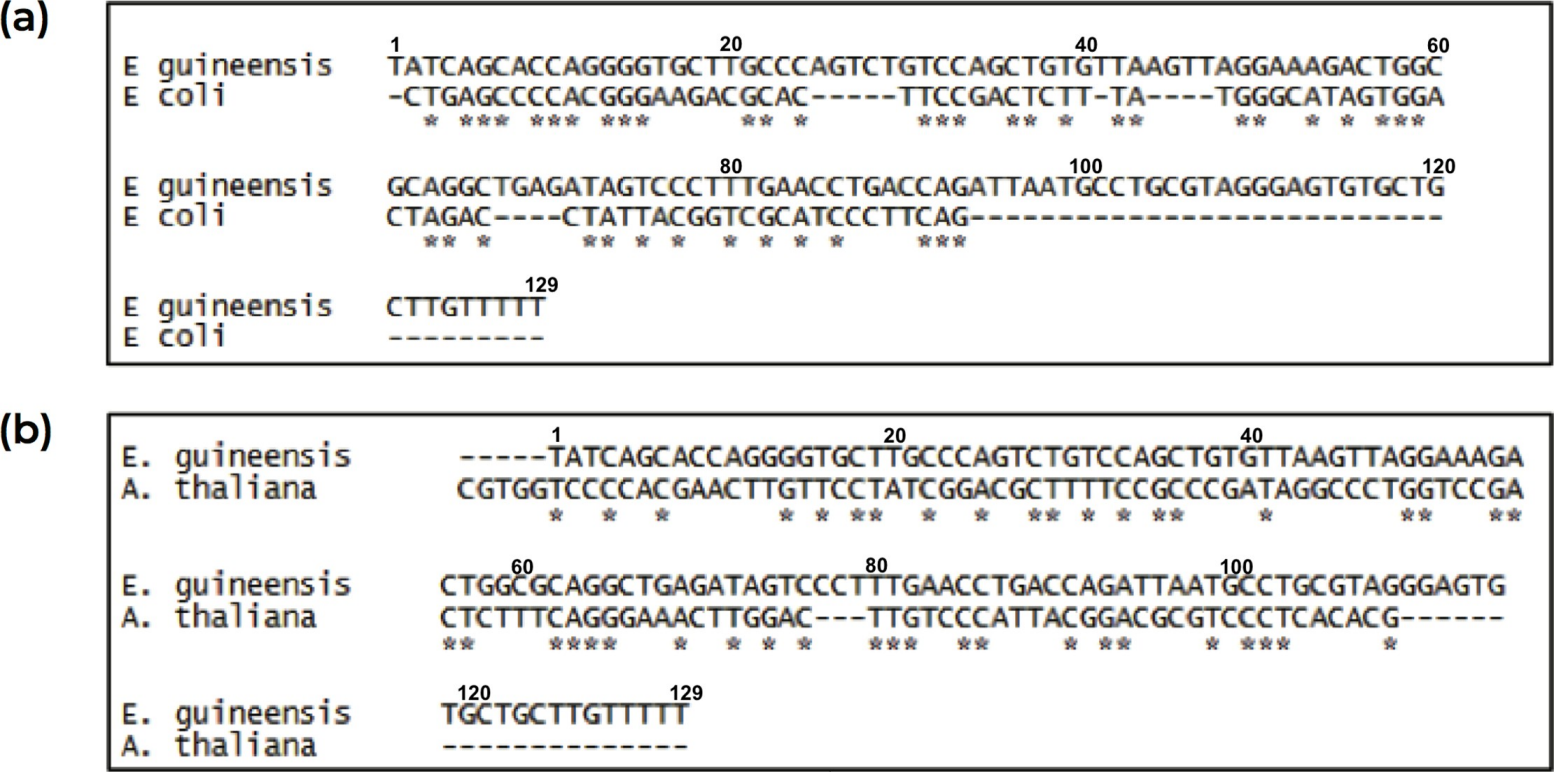
**(a)** An alignment of putative TPP riboswitch sequence of *E*. *guineensis* with conserved TPP aptameric region of *E*. *coli* **(b)** An alignment of putative TPP riboswitch sequence of *E*. *guineensis* with conserved TPP aptameric region of *A*. *thaliana* [27]. The conserved sequence is marked with (*).

While some of the aforementioned search methods for riboswitches contain a certain amount of structural consideration, it is important to put a considerable emphasis on structure without neglecting sequence conservation. When the putative TPP riboswitch sequence was compared with the conserve aptameric domain of *E*. *coli* TPP riboswitch shown in Fig 5(A), it was very conclusive that the aptamer region was well-conserved inter-species with 71% similarity [37]. Yadav *et al*. (2014) [32] also proved that the aptamer length of TPP riboswitch in several plants species was between 77 to 130 bp. For the past decade, conserved sequences and architecture for TPP motifs were exceedingly similar across all species and they are highly conserved in terms of their sequence and structure [10 and 36]. Fig 5(B) shows an alignment of conserved putative TPP riboswitch sequence of *E*. *guineensis* and *A*. *thaliana*. Alignment of putative TPP riboswitch of *E*. *guineensis* with same domain and different domain suggests that the sequence is conserved across species.

Above all, the identification of TPP riboswitch motif in oil palm was successfully conducted primarily by using several detection web based databases. These databases implemented two important features during the search. The cardinal step is the search of highly conserved region of the aptamer which consequently characterises the riboswitch family. Secondly, it also codes with algorithm that can predict the secondary structure and its stem loop formation. The prediction works is based on basis of the matches of new input with the known established structure. All in all, this so called molecular dynamic is seen to be a stepping stone in the structural study of riboswitch and further evaluation on the functionality of identified motif needs to be carried out.

### *In vitro* study: Analysis of ligand binding of TPP riboswitch in oil palm

#### Amplification of targeted putative *thiC* TPP riboswitch gene fragment

**Fig 6:**
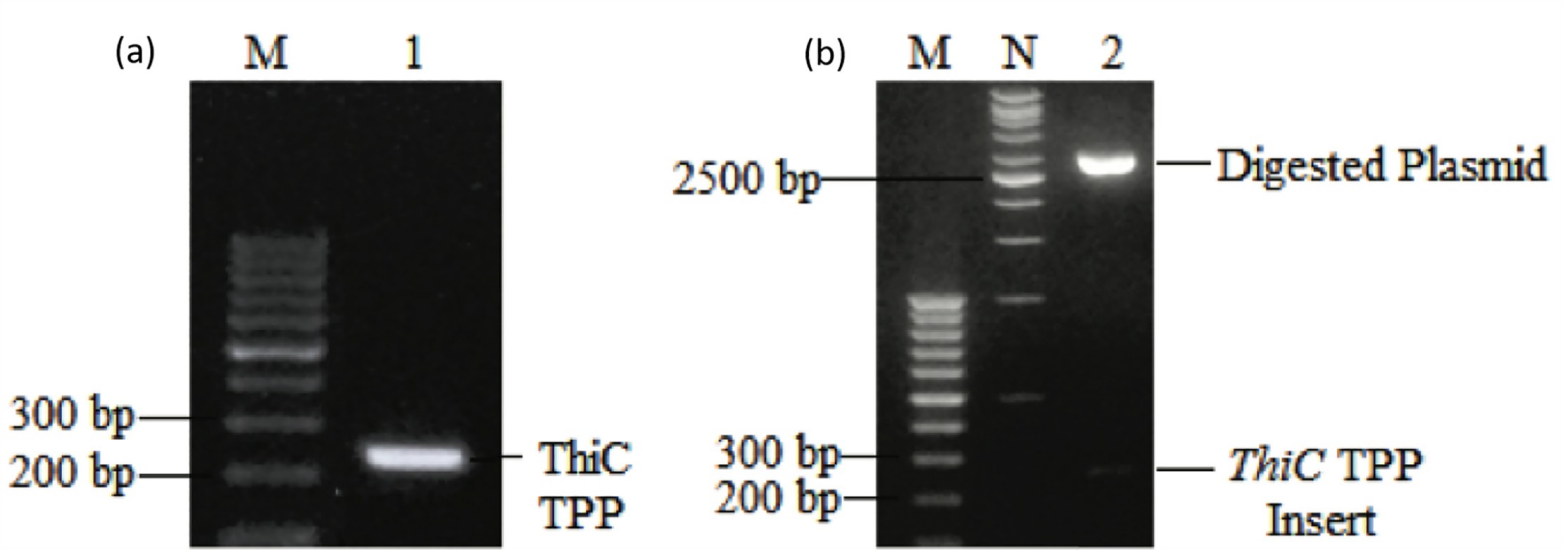
**(a)** Successful amplification of putative TPP riboswitch using primer designed. Lane 1 represents the amplified fragment at 242 bp. **(b)** Plasmid digestion reaction showing the recovery of plasmid at 3015 bp and the insert size at 242 bp (Lane 2). Lane M represents 100 bp DNA ladder and Lane N represents 1kb DNA ladder.

The putative TPP riboswitch was successfully amplified at specific sizes 242 bp, cloned and the sequence had been confirmed as shown in Fig 6.

Sequencing was then performed to confirm the sequence of the transcript (S2 Appendix). The sequencing result displayed a high similarity index to putative TPP riboswitch with 100% sequence similarity and E-value of 2e-71.

#### Analysis of ligand binding to putative *ThiC* Thiamine PyroPhosphate (TPP) riboswitch in oil palm

*In vitro* transcription was carried out to synthesise the targeted RNA sequence that was postulated to be a TPP riboswitch. Analysis of ligand binding to thiamine pyrophosphate was carried out using isothermal titration calorimetry (ITC). This technique is used to study the interaction between biomolecules based on the direct measurement of heat change during the binding event. Isothermal titration evaluated the binding ability of TPP riboswitch with its ligand through the measurement of heat change between TPP ligand and TPP riboswitch. Although this technique is label-free, no limitation on molecular weight, and can be considered as one of the most accurate techniques to evaluate the equilibrium constant, it has not been widely used in RNA binding interactions [38]. Several parameters were obtained including stoichiometry (n), binding affinity (K_d_), free energy change (ΔG), enthalpy change (ΔH) and entropy change (ΔS) [39]. In this context of study however, the focus was put on the binding activity which can be evaluated from the binding affinity (K_d_).

**Fig 7:**
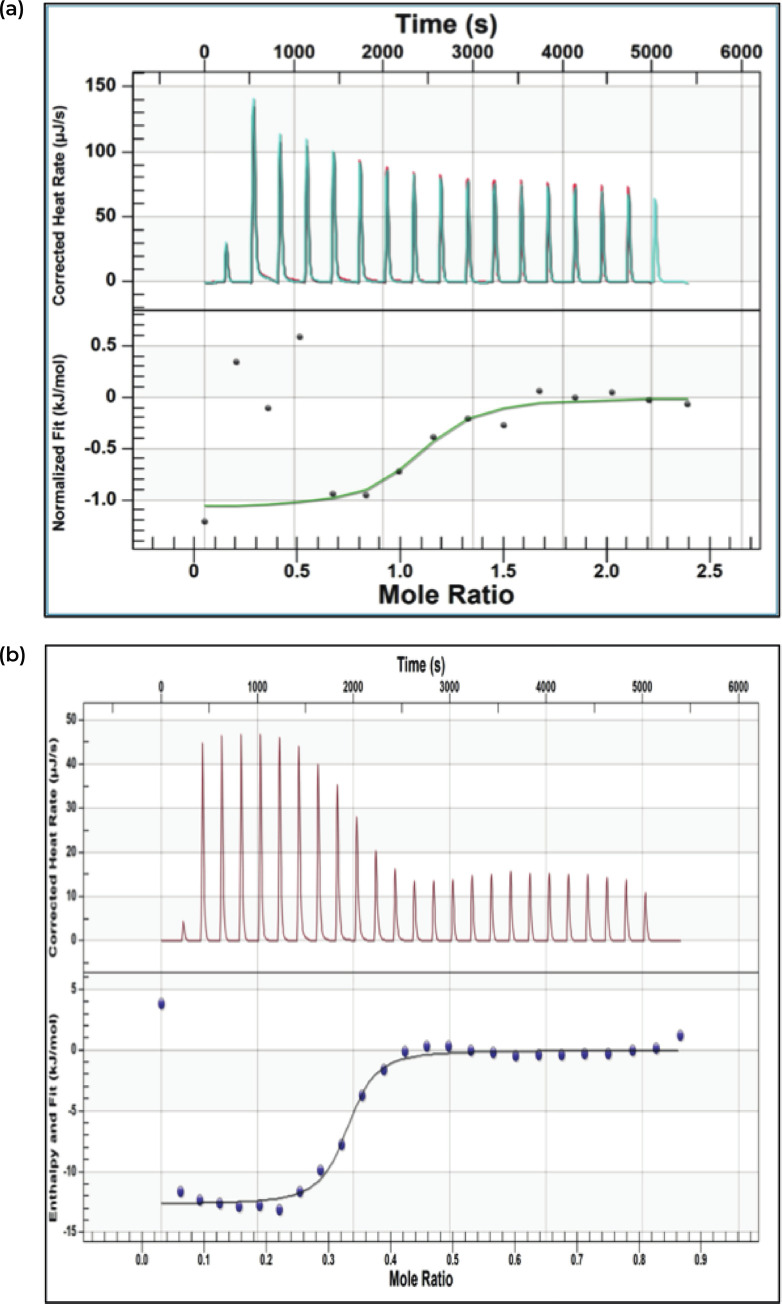
ITC analysis of (a) TPP ligand (100mM) with putative TPP riboswitch RNA (10 mM) at ratio of [10:1] (b) TPP ligand (30mM) with putative TPP riboswitch RNA (10mM) at ratio of [3:1].

The *thi*-box riboswitch regulates gene expression in response to the intracellular concentration of thiamine pyrophosphate (TPP) in archaea, bacteria, and eukarya [40]. The binding activity of putative TPP riboswitch and its ligand was investigated during this analyses. The determination of concentration for micromolecule and its ligand is the primary step in running ITC experiment. This can be obtained by simulating the thermogram with reasonable guesses for equilibrium constant, K and enthalpy change, ΔH. Fig 7(A) and 7(B) show the isotherm of putative TPP riboswitch-ligand at ratio of [1:10] and [1:3] respectively. Ligand was put in excess concentration to allow the titration activity reach its saturation phase. The first peak of titration activity gives out a small peak value. It is very common to have the anomaly result due to volumetric error that is caused by the backlash in motorised screw that drives the syringe plunger [41].

The construction of sigmoidal curve provided details on binding activity of putative TPP riboswitch of oil palm with enthalpy (ΔH) of -12.092 kJ/moL ± 0.8. Enthalpy change measures the energy content of the bond created and broken. The negative value indicates that the enthalpy is favouring the binding which usually dominated from the hydrogen bond or Van der Waals formation. The entropy (ΔS) of the titrant was 62.34 J/mol-K ± 4.7 which indicate the changes in hydrophobic interaction and conformational changes upon ligand binding. The stoichiometry indicates the number of binding site of micromolecule to its ligand. In this analysis, the stoichiometry of 0.906 ±0.011 was obtained which is in line with the hypothesis postulated that the number of binding site should be 1. Unlike protein, riboswitch is expected to have one binding site per molecule.

Finally, the equilibrium dissociation constant (K_d_) of the binding was found to be 0.178 nM ± 0.023. In thermodynamic theory of interaction, association constant (Ka) is reciprocal to dissociation constant (K_d_). Therefore, the lower the dissociation constant, the higher the association (K_a_); which indicates moderate binding strength. The change in heat capacity over the time testifies the binding activity of TPP riboswitch with its ligand that entails an important structural changes which relates to its function of controlled gene, by the induction of the RNA architecture [42]. As for the second targeted fragment (primer TPP2), the binding activity was said to be none since it produced linear heat rate activity. The data binding strength were compared with previously known riboswitch binding activity in other organisms. Evidently, up until today, the binding activity of riboswitch in other plants using isothermal titration is not available, therefore comparison of isotherm can only be done with other type of microorganisms (Table 1). Most widely studied organism for RNA-ligand interaction is *E*. *coli* and *B*. *subtilis*. In this study, the binding strength was said to be low to moderate binding. Nevertheless, this work provides the first evidence of binding activity that occur at the identified putative TPP riboswitch transcript. Binding acitivity details as mentioned in S3 Table.

**Table 1:** Comparison of analysis of ligand binding of TPP riboswitch in *E*. *guineensis* as compared to other microorganisms.

Organisms	Riboswitch	Kd (nM)	Binding strength
*Elaeis guineensis*	*ThiC* TPP 1	0.178	Low
*E*. *coli*	*ThiM*	8.43E-9	High
*L*. *rhamnosus*	*preQ1*	0.81E-9	High
*B*. *subtilis*	*ThiM*	3E-6	Moderate to high

### *In vivo* study: Gene expression profiling of *ThiC* and metabolite quantification

#### *ThiC* gene expression analysis upon exogenous thiamine application

RNA of oil palm seedlings was extracted on day 0, 1, 2 and 3 post treatment represented by D_0_, D_1_, D_2_ and D_3_ respectively. Application of exogenous thiamine was directly applied as it could be absorbed directly by the plant as it was transported through xylem and phloem in acro-petal and basi-petal directions [8 and 43].

#### Analysis of *ThiC* gene expression in oil palm upon exogenous application of thiamine

**Fig 8:**
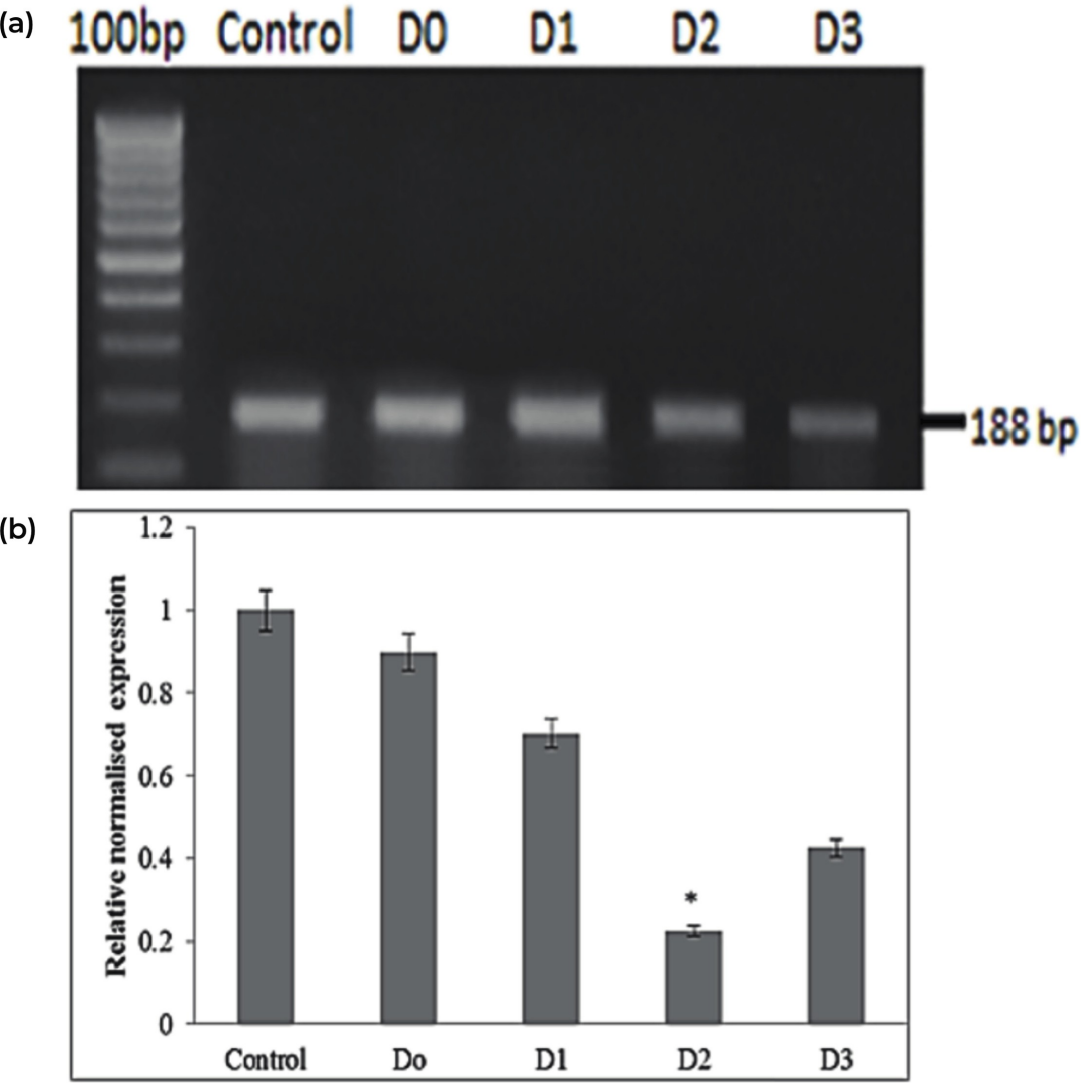
**(a)** PCR amplification *ThiC* gene fragment with descending band intensity. Lanes represent Control, Day 0, 1, 2 and 3 post-treatments. **(b)** Relative normalised expression of *ThiC* gene fragment in thiamine treated oil palm seedlings via qPCR analysis at Day 0, 1, 2 and 3 post-treatments. Data presented are the mean ± standard deviation of three replicates with significant differences of (p <0.05) using Student’s t-test.

Analysis of expression of *ThiC* gene was carried out by using quantitative real-time PCR (qPCR). In this study, the hypothesis is that the application of excess thiamine downregulates the *ThiC* gene expression. The decrease is due to the presence of riboswitch in the *ThiC* gene. As the application of exogenous thiamine has led to the presence of excess total thiamine content in the cells; which eventually leads to the formation of excess TPP. This is in turn binds to the TPP riboswitch that will down-regulates the expression of *ThiC* gene. Fig 8 shows the relative quantity of *ThiC* gene expression in oil palm seedlings throughout the 4 days’ treatment. The details of *ThiC* gene expression is summarised in S4 Table.

A similar analysis was done in year 2008 by Kong *et al*. [44] as they found that when *Arabidopsis thaliana* was treated with 100 mg/L of exogenous thiamine, the expression of *ThiC* gene was down-regulated one day after treatment and the expression intensity was maintained at low level during day 6, post treatment [44]. Extended study by Bocobza *et al*. (2008) [30] found that upon the increase of intracellular TPP concentration in *Arabidopsis* sp., TPP would bind to the riboswitch causing the conformational changes of its structure. This causes the splice site to be exposed in the 3’ end of *ThiC* gene. Following that incident, unstable 3’ UTR transcripts were produced resulting the decrease of *ThiC* protein level and consequently downregulate the biosynthesis of TPP in total.

A study on effect of exogenous thiamine on *Chlamydomonas reinhardtii* was conducted by the application of 10 μM of thiamine for 6 hours. After the addition of thiamine at 0 hour, it was found that the *ThiC* gene transcript decreased consistently until 6 hours post treatment [31]. Previous studies stated that TPP-binding riboswitches were responsible in controlling the thiamine biosynthesis in both *A*. *thaliana* and *C*. *reinhardtii* [31 and 45]. By comparing the *ThiC* gene expression of oil palm to both organisms, the prediction that TPP-riboswitch may also be present in the *ThiC* gene of oil palm and involved in controlling thiamine biosynthesis pathway is highly supported.

#### Quantification of thiamine metabolite through High Performance Liquid Chromatography (HPLC)

**Fig 9:**
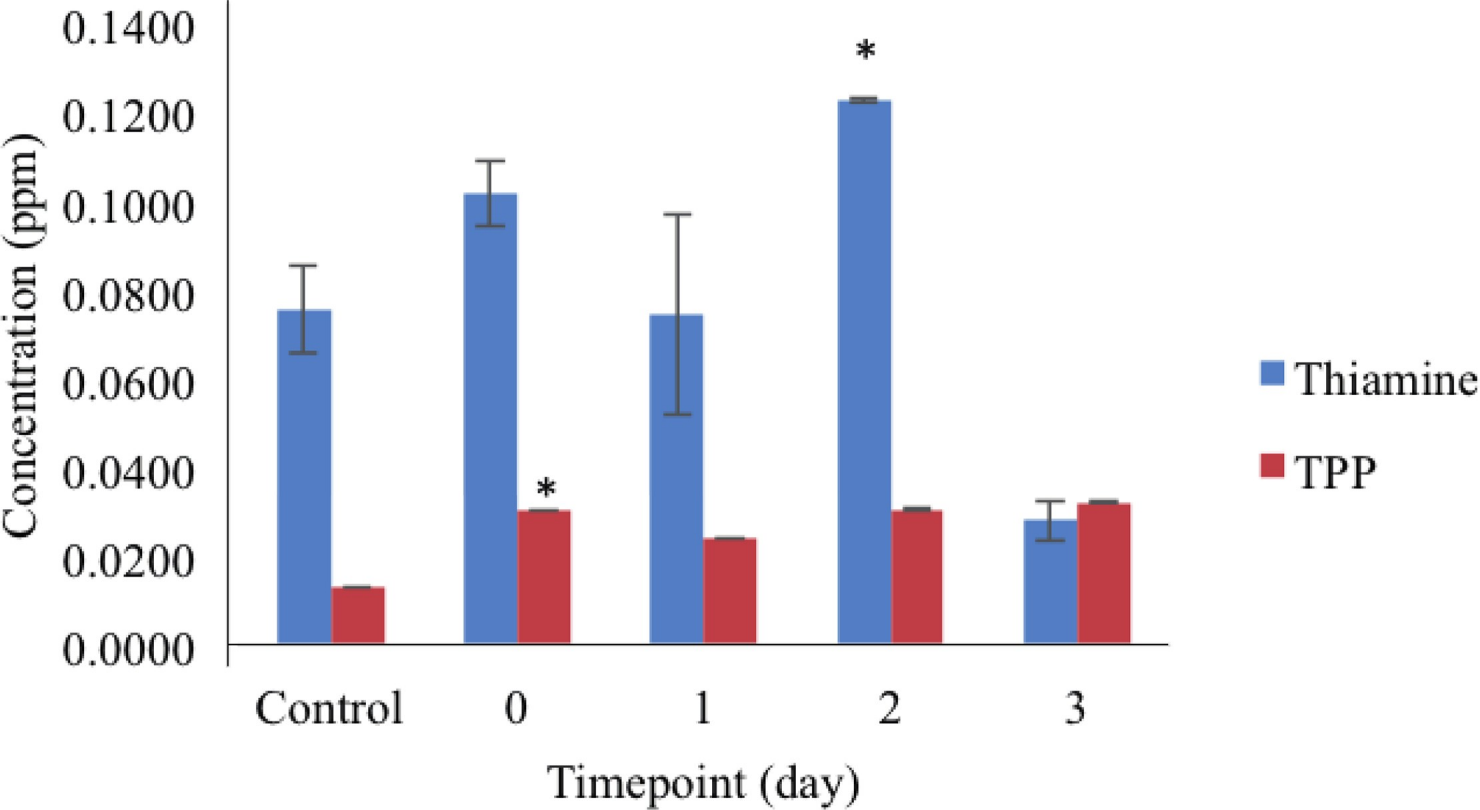
Quantification of Thiamine PyroPhosphate (TPP) in control (no thiamine) and thiamine treated oil palm seedlings at Day 0, 1, 2 and 3 post-treatments. Data presented are the mean ± standard deviation of three replicates with significant difference (*p* <0.05; Tukey’s multiple comparison test).

Thiamine metabolites namely thiamine and thiamine pyrophosphate (TPP) were measured through fluorescence detection instead of UV detection, as thiamine metabolites were unstable and sensitive to UV light which may cause degradation of metabolites during the analysis [46]. Analysis of metabolites via HPLC showed the concentration of thiamine decreased post thiamine treatment while TPP metabolites increased post thiamine treatment as shown in Fig 9. The increase of TPP content only occurred after one day post treatment might due to the time needed for the pool of thiamine to be converted into the active form, TPP. When exogenous thiamine was applied to oil palm seedlings, the metabolites were absorbed and entered thiamine biosynthesis pathway that increased the concentration of thiamine in the plant [47 and 31].

The decline in thiamine and increase in TPP metabolites could be related to the phosphorylation of thiamine into TPP, which is the active form of thiamine. The presence of excess TPP led to the binding to the TPP riboswitch that eventually caused the down regulation of *ThiC* gene expression. Negative correlation between metabolite content and *ThiC* gene expression suggests the negative feedback regulation conducted by TPP riboswitch present in the *ThiC* gene as previously reported by (Bocobza and Aharoni, 2014) [3]. The negative correlation between *ThiC* gene expression and thiamine content was also displayed in *Cassava* sp. as it is postulated that the B_1_ production was controlled by partial feedback regulation [2]. Details of quantification of metabolites were summarised in S5 Table.

## Conclusions

This study describes the identification of TPP riboswitch in oil palm through bioinformatics and experimental approaches. The analysis revealed the position, segment length, secondary structure and derivatization of its conserve region. The putative TPP riboswitch in oil palm (*Elaies guineensis*) was found to be located at the 3’ untranslated region of the non-coding *ThiC* gene with 192 nucleotides in length. Analysis of the gene of interest using numbers of riboswitches web tools showed more than 95% detection similarities. The conserved aptameric region of the riboswitch made up of 92 nucleotides based on comparative gene studies with previously identified aptamer domain. The aptamer region was seen to be conserved inter-species. The secondary structure of TPP riboswitch folded into stem-loop structure manifested with stem (P1-P5) with minimum free energy of -12.26 kcal/mol. Identification of putative TPP riboswitch by using computational methods can pave the way for further exploitation of this motif for better understanding of the regulation of thiamine biosynthesis pathway in oil palm.

Isothermal titration calorimetry analysis carried out in this study has revealed the binding ability of TPP riboswitch with its ligand through the measurement of heat change. The sigmoidal curve obtained from this analysis provided the details of the binding activity of the putative TPP riboswitch of oil palm with enthalpy (ΔH) of -12.09 kJ/moL, entropy (ΔS) of 62.34 J/mol-K, stoichiometry of 0.906 and equilibrium dissociation constant (K_d_) of 0.178 nM. The binding strength was said to be low to moderate binding. The change in heat capacity over the time verified the binding activity of TPP riboswitch with its ligand that entails an important structural change which related to its function of controlled gene, by the induction of the RNA architecture.

Analysis of *ThiC* gene expression upon exogenous application of thiamine suggested that thiamine biosynthesis in oil palm can be effectively regulated by the physiological concentrations of the vitamin at 50 mM with down regulation of *ThiC* gene expression of fivefold post thiamine treatment. Metabolites analyses revealed the decrease in thiamine content while increase in TPP content, supporting the prediction of the presence of the TPP riboswitch in oil palm *ThiC* gene.

A type of RNA that does not code for any protein yet possesses a direct function can be found abundantly in nature. Advantages of RNA regulation compared to DNA regulation system is that, a rapid response can be achieved since the target is mRNA. Specific detection of bases can be made since it is a conserved region with small number of bases. Current research showed that most detected riboswitch is only identified at bioinformatics level, therefore verification on this finding through experimental approaches need to be done.

In this study, identification and characterisation of TPP riboswitch in oil palm have been described. However, further studies need to be done especially on the examination on conformational changes of putative TPP riboswitch via probing method. This method is an extension of metabolite binding study as it tells us about the conformational changes that occur in a greater detail. The mechanism of action of the TPP riboswitch could also be determined. Other than that, studies on specific bases in the riboswitch will lead to the understanding of the structural and biological function of TPP riboswitch.

## Supporting information

S1 Appendix Gene sequence: *ThiC* gene of oil palm. (DOCX)

S2 Appendix Sequencing results. (DOCX)

S1 Text Preparation of media. (DOCX)

S2 Text Preparation of mobile phase for elution of high performance liquid chromatography. (DOCX)

S3 Text General information of chromatography setting. (DOCX)

S1 Table List of primers. List of primers used for amplification of putative TPP riboswitch.
(DOCX)

S2 Table List of primers used during the analysis of *ThiC* gene expression upon application of exogenous thiamine. *Actin* and *Beta-tubulin* were used as reference genes.
(DOCX)

S3 Table ITC analysis. (DOCX)

S4 Table Analysis of gene expression of *ThiC* gene. (DOCX)

S5 Table Quantification of metabolites. (DOCX)

S1 Fig Chromatography report: Retention time. (DOCX)

S1 Raw images (PDF)
