# Supplementary material for: Identification and characterisation of thiamine pyrophosphate (TPP) riboswitch in Elaeis guineensis
Source: PLoS One. 2020 Jul 29;15(7):e0235431. doi: 10.1371/journal.pone.0235431 (PMC7390266; doi:10.1371/journal.pone.0235431)
Supplement: S1 Appendix — (DOCX) [file pone.0235431.s001.docx]

**S1 Appendix. Gene sequence: *ThiC* gene of oil palm.**

**>XM_010924818.2 PREDICTED: Elaeis guineensis phosphomethylpyrimidine synthase, chloroplastic (LOC105046270), transcript variant X9, mRNA**

ATCCCAATCAAACGGCCCTAACATCAATTCTCCTCCCAGCCTCCCTTCCCACCTCTTCCCGTTATCATCCGCTGCAACCTGCTCCTGTGTTCCCCTCTCTCTCGTTCATGGTTCTAGGGTTTCCAACGAACAACAAATACCTAAATTTTCCCTCAACCAAATCATGCCTGCATCCATCGAATTCCTTCAATCCATCACTCCTCGCAACTTCCACCCCAATCCGAACTCTTTTTCCAAGGAATCCACCAGGAGATCGATCCTCTTTCCTTATCGATCGAGAGAATACTCCATAGAACCACCTCGCTTCTCCAAGAACCCATCTGCTTCCTTCTCATCCATCCACGCGAGGCATAAAGGCAGGGTCTTTGTAGAGGGATTCGATGCCTGGGGAGTTCTCCTGAGGAGTCGGAGATGGGCGGAGGCCACAAGAATCCGAGCCAACAGCTCCTGCGAGCGGGACAGCGACTCCAAGGCCGGTGCCTCCCCCGAGAAGAAGAACACAGAGAGCAGCCCGCCCACAAATAACAAAGGCATCAACAAGCCCTCCAGTCCTCCTCCTCCTTCTTCTTCTTCGTCATCGTCGTCGTCGTCACCGCGTCGGGAGAAGAAGTGGAAGGGTGGGTGGTGGAAGGGTGGGAGGTGGCAGTGGCAGCCGATAATCCAGGCCCAGGAGATCGGGGTGCTCCTGCTGCAGCTGGGAATTGTGATGTTCGCCATGCGACTGCTCCGGCCGGGGGTTCCGCTGCCGGGTTCGGAGCCGAGGACCCCGACAACCTACATCAGCGTGCCGTTCAGCGACTTCTTGAGCAAGATCAACAAGGATCAGGTGCAGAAGGTGGAGGTTGATGGGGTTCATCTCATGTTTAGGCTGAGGTCGGACGCGGAAAGTGCGGAGGCGGAGACAGGTAGGGGGAGTAGGTCGCAGGAGGCCGAGGCTTTGATCAGAGGCGTGCCCCCCACAAAAAGAATCGTGTACACCACTACCCGTCCGGGTGATATAAAGACTCCCTATGAGAAGATGCTGGAGAACCAGGTAGAATTTGGCTCGCCGGACAAGCGTTCTGGCGGTTTCTGGAATTCTGCTCTGATAGCTCTTTTCTATATAGCTTTACTTGCTGCAGTGCTGCAGCGCTTCCCGATAAGTTTTTCCCAGCATGCAGCGGGGCAGTTGAGGAACCGTAAATCTGCTGGTTCTGGTGGAGTGAAAGCATCTGAACATGCTGATATAGTCACTTTTGCAGATGTAGCTGGTGTAGATGAGGCAAAAGAAGAGCTGGAAGAAATTGTGGAATTCCTTAGAAATCCAGACAGATATATACGTCTTGGTGCTCGTCCTCCTCGAGGGGTTTTGTTGGTGGGTCTTCCTGGAACAGGTAAGACACTTTTAGCAAAAGCTGTAGCTGGAGAAGCAGAAGTTCCCTTTATAAGTTGTTCTGCAAGTGAATTTGTCGAATTGTATGTGGGTATGGGAGCCTCCCGTGTTCGGGATCTATTTGCAAGGGCAAAGAAGGAGGCACCATCCATCATTTTTATTGATGAGATAGATGCTGTGGCAAAAAGCCGTGATGGTCGATTTCGCATTGTCAGCAATGATGAGCGTGAGCAGACACTCAATCAGTTGCTTACAGAGATGGATGGATTTGATAGCAGCTCTGCTGTCATTGTCCTTGGAGCAACAAATCGTGCGGATGTCCTGGACCCTGCACTTCGTCGTCCTGGAAGATTTGATCGTGTGGTGATGGTGGAAACTCCTGATAGGTTTGGAAGAGAAGCCATTTTAAAGGTTCATGTAAACAAGAAGGAACTTCCTTTAGGAGATGATGTAGATCTTAGCGAAATTGCATCAATGACAACTGGTTTTACTGGAGCAGATCTAGCAAATTTGGTAAATGAAGCTGCTTTATTAGCTGGTAGAACAAGCAAAGTTGTTGTGGAAAAGATTGACTTTATCCTGGCAGTTGAACGTTCGATAGCTATGGCATCTATCCAACTGTCTCCTGTGGTGACTAACAAGAGCAACCATGCACCTTTCAAGTTGCCAAATACTCGCTTTTTACTTGGATTTGGATCAGCCAGGCATTCTTCAAATGTGTGGGATAAGGAAATGTACTACAATTCCATGAGATCCACGGTTAAAGCCTCCCTGACCTTTGATCATTCGATTGCTGAGTCTGCAAAAACCCAAAAGAAGAGGCACACTGTTGATCCTGCAGCTCCTGATTTTCTTCCATTTCCTTCTTTTGAAGAGTGCTTTCCGAAGAGTACCAAAGAATACAGGGAAATTGTTCATGAACAATCTGACCATGCCCTTAAAGTTCCATTTCGACGCATCCATCTGTCTGGGGAAGACCGGTACTTTGATACATATGATACCAGTGGCCCACAGAATATAAGTCCACGTGTGGGTCTTCCCAAAATAAGAACAGACTGGGTTGACAGGCGGGAAAGTTTGGGTGGACCAAGATACACCCAGATGTTCTATGCTAAACAGGGAATTGTAACAGAAGAGATGTTGTTTTGTGCCGCTCGTGAGAAACTTGATCCTGAATTTGTGCGGTCAGAGGTTGCACGTGGACGTGCTATAATTCCTTCTAATAAGAAGCATCTTGAGCTTGAGCCTATGATAATTGGGAGGAACTTCTTGGTCAAAGTGAATGCAAATATTGGGAACTCAGCTGTGGCGAGCTCTATCGAGGAAGAGGTGCATAAGCTTAAATGGGCAACAATGTGGGGAGCTGACACAGTCATGGATCTCTCAACAGGTCGTCATATCCATGAGACTCGTGAATGGATCCTACGAAACTCTGCGGTGCCGGTTGGGACTGTGCCTATCTACCAAGCACTGGAGAAAGTGAATGGCATTGCTGAAAATCTTAACTGGGAGATTTTTAGGGATACTCTGATTGAACAAGCCGAGCAGGGTGTTGACTATTTTACCATCCATGCTGGGGTGCTGCTTCGCTACATTCCTCTTACAGCAAAAAGAATGACCGGCATTGTTTCACGTGGAGGATCAATTCATGCAAAATGGTGCTTGGCTTATCACAAGGAGAACTTTGCTTATGAGCACTGGGATGATATACTCGACATATGCAATCAGTACGATGTGGCATTATCTATTGGCGATGGGTTGAGGCCTGGTTCAATTTATGATGCCAATGACACTGCTCAGTTTGCAGAACTTCTGACACAAGGGGAGCTGACACGTCGAGCATGGGAAAAGGATGTGCAGGTAATGAATGAAGGACCTGGACATATTCCAATGCACAAAATCCCAGAAAATATGGAAAAGCAATTGGAATGGTGTAATGAGGCACCTTTTTATACACTTGGTCCTCTAACGACTGATATTGCTCCTGGATATGATCACATCACCTCAGCAATTGGTGCTGCTAACATTGGAGCCCTTGGTACTGCACTTCTTTGTTATGTAACACCTAAGGAACACCTTGGGTTGCCGAATCGTGATGATGTGAAGGCAGGTGTGATATCATACAAGATAGCTGCCCACGCAGCTGATTTAGCAAAAGGTCACCCACATGCACAGGCATGGGATGATGCATTGAGCAATGCAAGATTTGAGTTTAGATGGATGGACCAGTTTGCTTTGTCTTTAGACCCTATGACTGCTATGGCTTTCCATGATGAGACCCTGCCATCAGAGGGTGCCAAGGTGGCACACTTCTGTTCTATGTGCGGGCCCAAATTTTTTCTATGAAAATAACAGAAGATGTGAGGAAGTATGCTGAGGAGCATGGATATGGGACAGTGGAGGAAGCTGTGAAACATGGAATGGATGCTATGAGTGCTGAGTTTCTGGCTGCAAGGAAAACTGTTAGTGGGGAACAACATGGCGAAGTTGGTGGAGAAATCTATGTGCCCGAGAGTTATGCTCATCAATCAAGGAACAATTAAAAATATTTTCATCATGGTATGCTTTGAAAGGAGCTAGAGCAGTGGGCGCTGTCTGGAGTCGTGATTGGTGAATAACAATCCTTAGATTGGGATGATGTGGGCAGGACTGGCTGAGAAAGTCCCTATGAACCTCAACAGGATGATACCTGCAGAGGGAGGGTGCAATTGTTTGTTTGTTGCTTCTGTTTTTTGGGTGATTGCTCAGGGAGTTCTCCAAGCGAGCATCTAACTTAGATTATTTCCAAGTGCACCAATCTTGCAGTCATTCGTGTCTGGGAAGTAATGAAAATTGCTTTCAATTTCCTCTAATTATTTTGCTTTTCATGGTTGGAGGAGTTTGTATCTTAGTTCTGACAATGTTTGATGCATGTTTTATAATGCCCATTTCGGCTATTGTAGAATAGCTGTTGTCAGAATTAAGCTACAATCAGTGGGTCTCCGTTCTTTCACGAGGGAGACAAAAGGAAAAAAGGGGGAAAGTAATGGGTTTTATAGGTGTGGTCTTGTGTCTTTTTCTCTTGCCCCATCTTTCCACAGGCTAATTATTATCAGCACCAGGGGTGCTTGCCCAGTCTGTCCAGCTGTGTTAAGTTAGGAAAGACTGGCGCAGGCTGAGATAGTCCCTTTGAACCTGACCAGATTAATGCCTGCGTAGGGAGTGTGCTGCTTGTTTTTGTTTTGCTTCTTTGCAGAAGGTGTTTTGAGGCAACATCAGCTCATGTTCATGCTGTAGCCGCTCTGCTTTTCATCTCCCATTAAGGCTGTACAAAATTACACTTCCAGATGGTTGTGACTTGATTCCTATGAATAGCAGGCAAGTCAGGTTAGAATTTCGGTAGCGAGGACTGGTTCTTAAGCGCTAAATAGAGGGGTGATTAGATCCCCTTTTGGGCAAGGCTCTCAAAAGCTATTGAGATATGGATGGATGCTGGAGACCTAACTTCTTTGGCTTTTATATTATATAAACCGCACGCTGTGTTGTGAATGATGAATGAGTTAATATAGCGCTAATTTATCCATGCCAAGTGGTGCTTTTAA

**Legend**

TPP riboswitch fragment (position 4404-4591)

Forward primer THIC TPP1

Reverse primer THIC TPP1
