# Supplementary material for: Identification and characterisation of thiamine pyrophosphate (TPP) riboswitch in Elaeis guineensis
Source: PLoS One. 2020 Jul 29;15(7):e0235431. doi: 10.1371/journal.pone.0235431 (PMC7390266; doi:10.1371/journal.pone.0235431)
Supplement: S2 Appendix — (DOCX) [file pone.0235431.s002.docx]

**S7 Appendix. Sequencing Results.**

**>2456546_5a_d1_ThiC TPP 1 (RNA-ligand interaction study)**

TTGGGGGTTCCGGGCTATTATTATCAGCACCAGGGGTGCTTGCCCAGTCTGTCCAGCTGTGTTAAGTTAGGAAAGACTGGCGCAGGCTGAGATAGTCCCTTTGAACCTGACCAGATTAATGCCTGCGTAGGGAGTGTGCTGCTTGTTTTTGTTTTGCTTCTTTGCAGAAGGTGTTTTGAGGCAA

**>ThiC F3 (Analysis of gene expression study)**

TCGCCGGCTTTCCCCGTCAAGGTCTACATCCTGGGCTCCCTTTATGGTTCCGATTTAGGGCTTTACTGCACCTCGACCGCCAGAAACTTGAGTAAGGTGATGGTTCACGTATCGTGTCATCTTCCTGATAGACGGTTTTTCGCCCTTTGACGTTTGAGTCCACGTTCTTTAATACTGGACTCTTGTTCCAAACTGGAACAACACTCAACCCTATCTCGCTCTATTCTTTTGATTTATAAGGGATTTTGCCGATTTCGGGCTATTGCTTAAAAAATGAGCTGATTTAACACATATTTAACGCGAATTTTAACAAAATATTAACGCTTACAATTTCCTGATGCGGCATTTTCTCCTTACACATCTGTGCAGTATTTCACACCACATCGGGTGGTACTTTTCTCGGAAATGTGCGCAGAAACCCCAATTGATTTACTATACAAAAAACGTGCAATTTTGCATCCGCTCATGAGTCAATAATCCCTGATAAAGGCTTTCATTACTTTTGGAAAAGGGAGCAGTATTAACAATCCGACATTTGCCGGGACCTCCTTAGTCCCATTTTTTGCGGAAATTCTGACTTGCTTGTTTTTGCTAACCCAATAAAGCTTGGTGAACGCTAAAAGAAGCCTAAAAACCGGTTGGAATGCCCCAAATGGGTTTTCCATCAAAGCTGGAATTTCAACAATTCGGAAAAATTCCTTGGAAAGCTTTTACTCCCCAATAAACGGTTTCCTATTGACCTGACCCGTTTTTAAGCTTCCCCCAAAAGGGGCCGCGA

| **Primer’s name** | **Gene** | | **Sequence similarity** | **E-value** |
| --- | --- | --- | --- | --- |
| THIC TPP F1 R1 | *Elaeis guineensis* phosphomethylpyrimidine synthase | | 100 % | 2e-71 |
| THIC F3 R3 | | *Elaeis guineensis* phosphomethylpyrimidine synthase | 99.2 % | 4e-38 |
