# Supplementary material for: Identification and characterisation of thiamine pyrophosphate (TPP) riboswitch in Elaeis guineensis
Source: PLoS One. 2020 Jul 29;15(7):e0235431. doi: 10.1371/journal.pone.0235431 (PMC7390266; doi:10.1371/journal.pone.0235431)
Supplement: S1 Text — (DOCX) [file pone.0235431.s003.docx]

**S1 Text. Preparation of Media.**

**Preparation of 3 % X gal**

0.15 g of X-gal was dissolved in 5 mL dimethyl formamide (DMF). It was stored at -20 °C.

**Preparation of 100 mM isoprppyl β-D-1- thiogalactopyranoside (IPTG)**

0.12 g of IPTG was dissolved in 5 mL sterile distilled water and filtered sterilised before being stored at -20 °C.

**Preparation of 100 mg/mL ampicillin**

0.5 g of ampicillin was dissolved in 5 mL sterile distilled water and filtered sterilised before being stored in aliquots at -20 °C.

**Preparation of 500 mL of LB broth**

12.5 g of LB broth was dissolved in in 500 mL sterile distilled water. It was autoclaved at 121 °C for 15 mins and stored at 4 °C.

**Preparation of 500 mL of LB agar**

18.5 g of LB agar was dissolved in in 500 mL sterile distilled water. It was autoclaved at 121 °C for 15 mins. The agar was allowed to cool down until the bottle can be held with hands. Then, 500 μL of each ampicillin, X-gal and IPTG was added inside the LB agar solution. Mix thoroughly. About 20 mL of LB agar was transferred into plastic petri dish (9 cm diameter). The plates were sealed using Parafilm and stored at 4°C.
