# Supplementary material for: Identification and characterisation of thiamine pyrophosphate (TPP) riboswitch in Elaeis guineensis
Source: PLoS One. 2020 Jul 29;15(7):e0235431. doi: 10.1371/journal.pone.0235431 (PMC7390266; doi:10.1371/journal.pone.0235431)
Supplement: S2 Text — (DOCX) [file pone.0235431.s004.docx]

**S4 Text. Preparation of Mobile Phase for Elution of High Performance Liquid Chromatography.**

1. Pre-mix sodium phosphate buffer (10 mM, pH 7.3) 100 mM of sodium phosphate monobasic (NaH_2_PO_4_) and 100 mM of sodium phosphate dibasic (Na_2_HPO_4_) were prepared. Pre-mix buffer of both were prepared with concentration of 10 mM with pH 7.3.
2. Methanol (100 %)

Both mobile phase buffers were filtered using Whatman nylon membrane filter, 0.2 μm, 47 mm (diameter). Then, the solution was degassed using sonicator for 10 mins.
