# Supplementary material for: Identification and characterisation of thiamine pyrophosphate (TPP) riboswitch in Elaeis guineensis
Source: PLoS One. 2020 Jul 29;15(7):e0235431. doi: 10.1371/journal.pone.0235431 (PMC7390266; doi:10.1371/journal.pone.0235431)
Supplement: S3 Text — (DOCX) [file pone.0235431.s005.docx]

**S5 Text. General Information of Chromatography Setting.**

Instrument : High performance liquid chromatography (HPLC)

Brand : Agilent Technologies

Model : 1200 Series

Column flow : 1.00 mL/min

Stop time : 10 min

Solvent A : 95 % Na_2_PO_4_

Solvent B : 5 % Methanol

Temperature : not controlled

Column : C18 (SGE Wakosil 5 µm)
