# Supplementary material for: Identification and characterisation of thiamine pyrophosphate (TPP) riboswitch in Elaeis guineensis
Source: PLoS One. 2020 Jul 29;15(7):e0235431. doi: 10.1371/journal.pone.0235431 (PMC7390266; doi:10.1371/journal.pone.0235431)
Supplement: S1 Table — List of primers used for amplification of putative TPP riboswitch. (DOCX) [file pone.0235431.s006.docx]

**S2 Table. List of Primers.** List of primers used for amplification of putative TPP riboswitch

| Enzyme | Primer’s name | Sequence (5’-3’) | Amplicon size (bp) | Ta (°C) |
| --- | --- | --- | --- | --- |
| Hydroxymethyl pyrimidine synthase | *ThiC*TPP 1 (F)  *ThiC*TPP 1 (R) | GGTGTGGTCTTGTGTCTT  CGGCTACAGCATGAACAT | 242 | 61 |
