# Supplementary material for: Identification and characterisation of thiamine pyrophosphate (TPP) riboswitch in Elaeis guineensis
Source: PLoS One. 2020 Jul 29;15(7):e0235431. doi: 10.1371/journal.pone.0235431 (PMC7390266; doi:10.1371/journal.pone.0235431)
Supplement: S2 Table — Actin and Beta-tubulin were used as reference genes. (DOCX) [file pone.0235431.s007.docx]

**S3 Table. List of primers used during the analysis of *ThiC* gene expression upon application of exogenous thiamine. *Actin* and *Beta-tubulin* were used as reference genes.**

| **Enzyme** | **Primer’s name** | **Sequence (5’-3’)** | **Amplicon size (bp)** | **Ta (°C)** |
| --- | --- | --- | --- | --- |
| Actin | *Actin* (F)  *Actin* (R) | CTTGCTCCAAGCAGCATGA  AGAAGCACTTCCGGTGCACG | 169 | 55 |
| Beta-tubulin | *Tubulin* (F3)  *Tubulin* (R3) | ACACGGCATAGATCCAACCG  TGGTTCCAGGCTCCAAATC | 147 | 61 |
| Hydroxymethyl pyrimidine synthase | *ThiC* (F3)  *ThiC* (R3) | AATGAAGGTCCAGGGCAT  GCTGAGGTGATGTGATCA | 188 | 60 |
