# Supplementary material for: Identification and characterisation of thiamine pyrophosphate (TPP) riboswitch in Elaeis guineensis
Source: PLoS One. 2020 Jul 29;15(7):e0235431. doi: 10.1371/journal.pone.0235431 (PMC7390266; doi:10.1371/journal.pone.0235431)
Supplement: S3 Table — (DOCX) [file pone.0235431.s008.docx]

**S8 Table. ITC analysis.**

**Binding activity details**

| **Parameters** | **Duplicate** | | | **Average** | **SD** |
| --- | --- | --- | --- | --- | --- |
|  | **1** | **2** | **3** |  |  |
| Kd (nM) | 0.1895 | 0.1948 | 0.1519 | 0.178733333 | 0.023388958 |
| Binding site (n) | 1.041 | 0.312 | 0.866 | 0.906333333 | 0.11970937 |
| ∆H (kJ/mol) | -10.077 | -12.54 | -12.66 | -12.09233333 | 0.081349155 |
| ∆S (J/mol-K) | 67.71 | 48.82 | 50.49 | 62.34 | 4.72492328 |
