# Supplementary material for: Identification and characterisation of thiamine pyrophosphate (TPP) riboswitch in Elaeis guineensis
Source: PLoS One. 2020 Jul 29;15(7):e0235431. doi: 10.1371/journal.pone.0235431 (PMC7390266; doi:10.1371/journal.pone.0235431)
Supplement: S4 Table — (DOCX) [file pone.0235431.s009.docx]

**S9 Table. Analysis of gene expression of *ThiC* gene.**

| **Replicate** | **Time**  **point** | **Ct ThiC** | **Ct (reference gene)** | **ΔCt treated** | **Δ ct control** | **ΔΔCt (ΔCt treated-ΔCt control** | **Fold difference in target genes relative to control** |
| --- | --- | --- | --- | --- | --- | --- | --- |
| 1 | ThiC-S0 | 25.15 | 27.28 | -2.13 | -1.95 | -0.18 | 0.8827 |
|  | ThiC-S1 | 27.41 | 29.08 | -1.67 | -1.95 | 0.28 | 1.2142 |
|  | ThiC-S2 | 29.00 | 33.42 | -4.42 | -1.95 | -2.47 | 0.1805 |
|  | ThiC-S3 | 26.83 | 31.92 | -5.09 | -1.95 | -3.14 | 0.1134 |
| 2 | ThiC-S0 | 25.01 | 27.32 | -2.31 | -1.95 | -0.36 | 0.7792 |
|  | ThiC-S1 | 26.72 | 30.55 | -3.83 | -1.95 | -1.88 | 0.2717 |
|  | ThiC-S2 | 29.22 | 32.19 | -2.97 | -1.95 | -1.02 | 0.4931 |
|  | ThiC-S3 | 27.22 | 30.52 | -3.30 | -1.95 | -1.35 | 0.3923 |
| 3 | ThiC-S0 | 25.34 | 27.4 | -2.06 | -2.11 | 0.05 | 1.0353 |
|  | ThiC-S1 | 27.10 | 29.90 | -2.8 | -2.11 | -0.69 | 0.6199 |
|  | ThiC-S2 | 20.06 | 33.01 | -12.95 | -2.11 | -10.84 | 0.0005 |
|  | ThiC-S3 | 27.71 | 30.20 | -2.49 | -2.11 | -0.38 | 0.7684 |
