# Supplementary material for: Identification and characterisation of thiamine pyrophosphate (TPP) riboswitch in Elaeis guineensis
Source: PLoS One. 2020 Jul 29;15(7):e0235431. doi: 10.1371/journal.pone.0235431 (PMC7390266; doi:10.1371/journal.pone.0235431)
Supplement: S5 Table — (DOCX) [file pone.0235431.s010.docx]

**S10 Table. Quantification of metabolites.**

**Thiamine**

| Time point (day) | Duplicate (ppm) | | | Average | SD |
| --- | --- | --- | --- | --- | --- |
|  | 1 | 2 | 3 |  |  |
| Control | 0.06867 | 0.0757 | 0.0827 | 0.0757 | 0.009921 |
| 0 | 0.0963 | 0.1015 | 0.1067 | 0.1015 | 0.007354 |
| 1 | 0.0902 | 0.0743 | 0.0584 | 0.0743 | 0.022486 |
| 2 | 0.1224 | 0.1227 | 0.1229 | 0.1227 | 0.000354 |
| 3 | 0.0249 | 0.0281 | 0.0313 | 0.0281 | 0.004525 |

**Thiamine pyrophosphate (TPP)**

| Time point (day) | Duplicate (ppm) | | | Average | SD |
| --- | --- | --- | --- | --- | --- |
|  | 1 | 2 | 3 |  |  |
| Control | 0.013 | \| 0.0131 \| \| --- \| | 0.01305 | 0.01305 | \| 7.071E-05 \| \| --- \| |
| 0 | 0.0302 | \| 0.0302 \| \| --- \| | 0.0302 | 0.0302 | 0.000E+00 |
| 1 | 0.0241 | \| 0.024 \| \| --- \| | 0.02405 | 0.02405 | 7.071E-05 |
| 2 | 0.0306 | 0.0302 | 0.0304 | 0.0304 | 2.828E-04 |
| 3 | 0.0321 | 0.0322 | 0.03215 | 0.03215 | 7.071E-05 |
