# Supplementary figures and images for: Identification and characterisation of thiamine pyrophosphate (TPP) riboswitch in Elaeis guineensis
Source: PLoS One. 2020 Jul 29;15(7):e0235431. doi: 10.1371/journal.pone.0235431 (PMC7390266; doi:10.1371/journal.pone.0235431)

**S6 Fig. Chromatography Report: Retention time.**

**
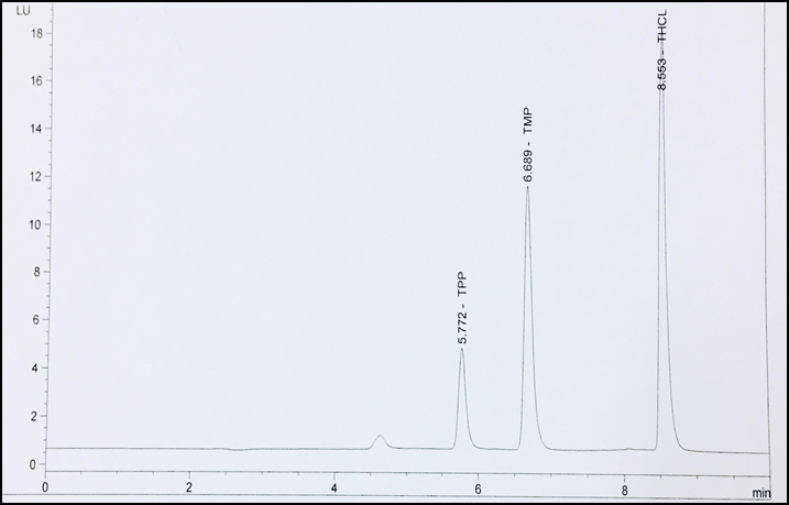
**

**

**

Supplement: S1 Fig — (DOCX) [file pone.0235431.s011.docx]
