# Supplementary material for: Identification and characterisation of thiamine pyrophosphate (TPP) riboswitch in Elaeis guineensis
Source: PLoS One. 2020 Jul 29;15(7):e0235431. doi: 10.1371/journal.pone.0235431 (PMC7390266; doi:10.1371/journal.pone.0235431)
Supplement: S1 Raw images — (PDF) [file pone.0235431.s012.pdf]

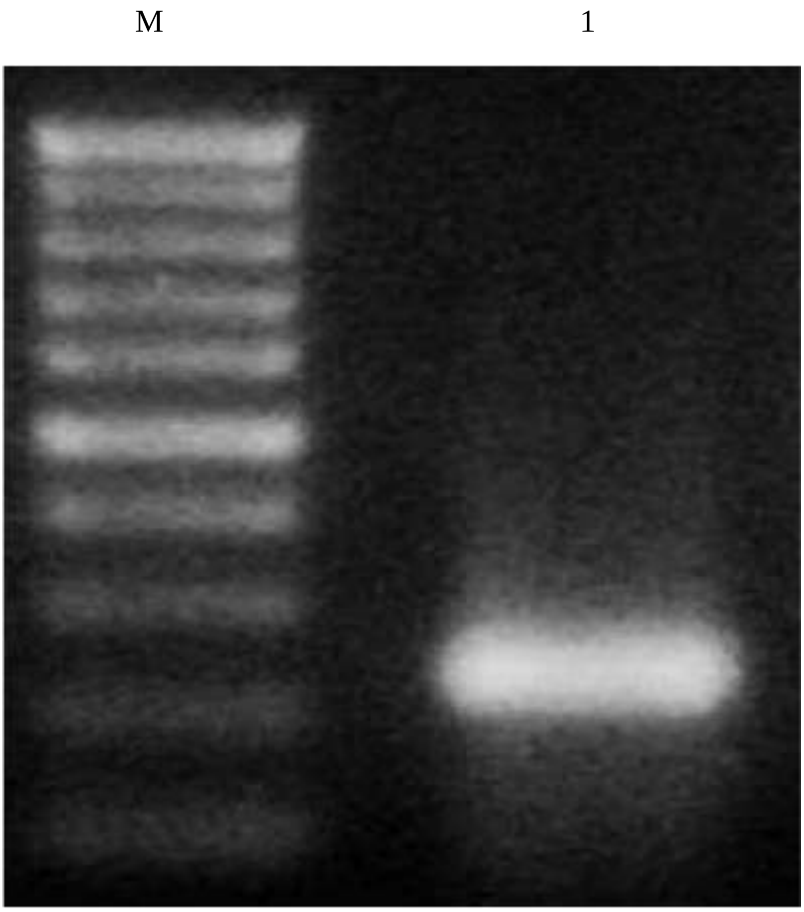

Lane M: 100 bp DNA ladder (New England Biolabs, England)

Lane 1: Amplified riboswitch fragment (242 bp)

Image capturing method: Gel Logic Pro Imaging System (Carestream Health, New York)

Figure 6(a)

M N 1 2 3

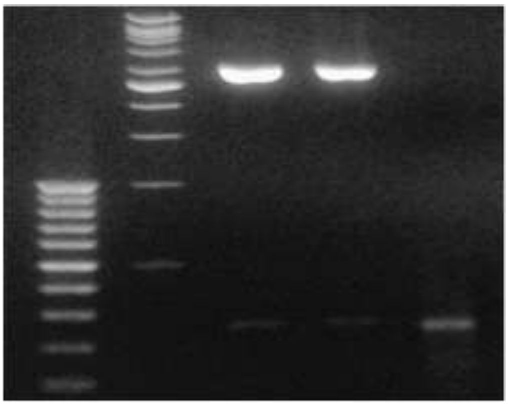

Lane M: 100 bp DNA ladder (New England Biolabs, England)

Lane N: 1 kb DNA ladder (New England Biolabs, England)

Lane 1: Digested plasmid at 3015 bp and the insert size at 242 bp (replicate 1)

Lane 2: Digested plasmid at 3015 bp and the insert size at 242 bp (replicate 2)

Lane 3: control insert size at 242 bp

Image capturing method: Gel Logic Pro Imaging System (Carestream Health, New York)

Figure 6(b)

Fig 6: (a) Successful amplification of putative TPP riboswitch using primer designed. Lane 1 represents the amplified fragment at 242 bp. (b) Plasmid digestion reaction showing the recovery of plasmid at 3015 bp and the insert size at 242 bp (Lane 2). Lane M represents 100 bp DNA ladder and Lane N represents 1kb DNA ladder.

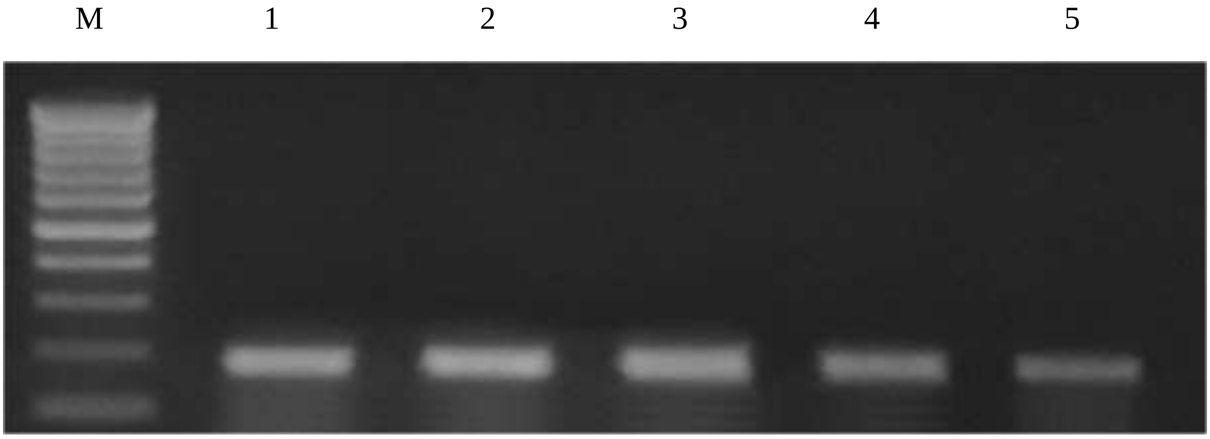

Lane M: 100 bp DNA ladder (New England Biolabs, England)

Lane 1: Amplified *ThiC* gene fragment (188 bp) non-treated thiamine

Lane 2: Amplified *ThiC* gene fragment (188 bp) Day 0

Lane 3: Amplified *ThiC* gene fragment (188 bp) Day 1

Lane 4: Amplified *ThiC* gene fragment (188 bp) Day 2

Lane 5: Amplified *ThiC* gene fragment (188 bp) Day3

Image capturing method: Gel Logic Pro Imaging System (Carestream Health, New York)

Figure 8(a)

Fig 8. (a) PCR amplification *ThiC* gene fragment with descending band intensity. Lanes represent Control, Day 0, 1, 2 and 3 post-treatments. (b) Relative normalised expression of *ThiC* gene fragment in thiamine treated oil palm seedlings via qPCR analysis at Day 0, 1, 2 and 3 post-treatments. Data presented are the mean ± standard deviation of three replicates with significant differences of ($p < 0.05$) using Student's t-test.
